# What Type of Consumption Induces or Alleviates Consumer Psychological Distress? Evidence From the COVID-19 Pandemic in China

**DOI:** 10.3389/fpsyg.2021.619303

**Published:** 2021-08-13

**Authors:** Yong Wang, Shuo Chen, Dandan Yang, Yang Li

**Affiliations:** Business School, Beijing Technology and Business University, Beijing, China

**Keywords:** COVID-19, psychological distress, consumption repression, consumption expectation, purchase behavior

## Abstract

Even though the coronavirus disease (COVID-19) has limited consumption, individuals continue to plan post-pandemic consumption activities to get rid of the stress caused by consumption repression. Building on Maslow’s theory of needs and Herzberg’s two-factor theory, our research categorizes consumption into fundamental (“must-have” products that fulfill the physical needs of individuals), hygiene (maintaining the security needs of consumers), and motivational consumption (enhancing well-being of individuals). Based on empirical data of purchase behavior and consumption expectation before, during, and after the pandemic in China, we identify how consumption repression induces psychological distress, *via* a sense of feeling threatened, lacking control, or lacking freedom, and how the expectation of future consumption alleviates that stress. Results show that fundamental consumption leads to psychological distress; hygiene consumption can both result in and reduce stress; and motivational consumption can reduce stress. Our findings provide new insights into the relationship between consumption and psychological distress through new theoretical formulations. The results can be applied by marketers attempting to understand purchase decision-making and by policymakers supporting both citizens and commerce during social emergencies.

## Introduction

Imagine how you would feel when you cannot purchase the products you want, and if your consumption is limited, what you would expect to see in your future shopping basket. The recent coronavirus (COVID-19) pandemic has become an obstacle for consumers attempting to purchase goods and services as usual, inducing psychological distress consequently (Elhai et al., 2020; Li et al., 2020). At the same time, consumers expect to purchase certain products when the pandemic is over; these expectations may help alleviate, to some extent, their current levels of stress (Galinsky et al., 2012; Hobbs, 2020). Recently, scholars paid attention to the psychological distress that elicited by COVID-19 (Paredes et al., 2021), and they were trying to identify approaches to reduce the stress (Waugh, 2020). According to previous research in stress regulation, consumption was one of the effective approaches to alleviate stress during COVID-19 (Chodkiewicz et al., 2020; Chenarides et al., 2021). Yet, our research focuses on the psychological distress induced by the consumption repression during COVID-19, and we propose that consumption repression in certain categories leads to different types of psychological distress, which in turn, can be alleviated by corresponding consumption expectations.

Our assumption is grounded in previous literature regarding the relationship between consumption and psychological distress (e.g., Gao et al., 2009; Kim and Rucker, 2012; Ma et al., 2019; Kim, 2020) and in theories of stress and coping (e.g., Lazarus and Folkman, 1984; Han et al., 2015). We contribute to current research by categorizing consumption into three types based on Herzberg’s two-factor theory and on Maslow’s hierarchy of needs: fundamental consumption, hygiene consumption, and motivational consumption (Herzberg, 2005; Huitt, 2007; Alfayad and Arif, 2017). Our study identifies the specific role played by each form of consumption in inducing or recovering from three types of psychological distress: a sense of feeling threatened, of lacking control, and of lacking freedom.

To the best of our knowledge, we are the first to compare consumption behavior and consumption expectation before, during, and after the COVID-19 pandemic. Our findings provide more comprehensive insight to understand the relationship between consumption and psychological distress, and moreover, we give solid support for marketers attempting to rearrange or reallocate products or services to boost post-pandemic sales and for government leaders seeking to effectively manage market supply to reduce psychological distress of consumers.

## Theoretical Development

### Consumption and Psychological Distress

Scholars have claimed that the consumption of certain products can lead to relevant feelings (Hobbs, 2020). For example, the purchase of luxury products may make consumers feel safe (Bettman and Fizsimons, 2013; Ma et al., 2019). Stockpiling larger quantities of food, such as grains, vegetables, and fruits, may ensure a lower level of negative emotion among consumers (Gibson-Smith et al., 2020). In the same way, the purchase or the expectation of purchasing certain products may influence, or even change, current feelings of consumers, because people who are feeling threaten could use products to complete or compensate the threaten part of the self (Gollwitzer et al., 1982; Broniarczyk et al., 1998; Lee and Shrum, 2013).

When consumers feel stressful, they would search for ways to cope with that stress (Duhachek, 2005). Two distinctive coping approaches have been defined: problem-focused and emotion-focused (Lazarus and Folkman, 1984). The former highlights ways to remove the stressor (Witte and Allen, 2000), whereas the latter emphasizes the importance of viewing things from alternative perspectives (Knoll et al., 2009). Problem-focused consumption may effectively diminish the sources of stress; for example, purchasing functional safety products may reduce the sense of being threatened (Griskevicius and Kenrick, 2013), or chose products that can increase consumer’s sense of power (Rucker and Galinsky, 2008). When consumers feel a lack of control, they increase spending on necessary products (Durante and Laran, 2016) or prefer products with boundaries, such as the designed places to be used or the recognizable logo (Cutright, 2012).

In contrast, emotion-focused consumption leads consumers to adopt compensatory, hopeful, or positive thinking (Tugade and Fredrickson, 2004; Shavitt et al., 2009). If consumers feel that they cannot solve the problems that cause stress, they sometimes turn to emotion-focused consumption for help (Duhachek and Iacobucci, 2005). For example, consumers search for compensatory consumption if they feel threatened (Mandel et al., 2017). They also consume more food to cope with stress (Richardson et al., 2015).

Abundant literature has supported the relationship between consumption and psychological distress. Scholars agreed that consumers feel stressed if their consumption of certain products becomes repressed, and the stress could be alleviated by consuming corresponding products (Tay and Diener, 2011). However, it still needs exploring what types of products can be used to reduce stress (Torres and Nowson, 2007; Durante and Laran, 2016). Therefore, we propose a new framework to match the types of psychological distress with the categories of products. Specifically, our study addresses the gap by investigating the influence of consumption repression in relation to six types of products or services. We divided the six types of consumption items into three categories: fundamental products, hygiene products, and motivational products. In contrast to previous studies that focused on stress caused by factors other than consumption, such as daily busy work (Burroughs and Rindfleisch, 2002), our current research discusses psychological distress elicited primarily by consumption repression.

### Consumption Categories and Stress Types

Scholars have explored varied ways to categorize products. In our study, we divide products by consumer motivation, an approach based on Herzberg’s two-factor theory and Maslow’s hierarchy of needs (Herzberg, 2005; Huitt, 2007). According to Maslow (1943), human behaviors are motivated by corresponding needs: physiological needs, security needs, social needs, esteem needs, and self-actualization needs. Consumers with different levels of need will be motivated to purchase corresponding products (Tay and Diener, 2011). Physiological needs are the basic needs of living, such as the need for food and water (Desmet and Fokkinga, 2020). On this level, individuals need to purchase these kinds of “must-have” products to fulfill the physical requirement for human survival (Boone et al., 2014), and we defined these kinds of products as “fundamental products.” Security needs are the psychological needs for safety (Desmet and Fokkinga, 2020). On this level, consumers would purchase products that make them feel safe and stable (Chen et al., 2015). Our research named these kinds of products as “hygiene products.” Social needs include needs for love and belongings, which can be fulfilled by a relationship with others. Esteem needs contain needs for respect, recognition, and competence, whereas self-actualization needs refer to needs for personal growth and meaning-making. Altogether, the last three levels of needs are correlated to the enhancement of well-being (Martela and Sheldon, 2019; Sirgy, 2019). Yet, we categorize the products that are being used to increase the well-being of individuals as motivational products.

The two-factor theory proposed by Herzberg et al. (1959) stated that some factors correlated to job content, such as challenging work, recognition, and responsibility, will make workers feel satisfied; whereas other factors that are correlated to the work environment or work relationships, such as salary and insurance, could only make workers not to feel dissatisfied. The former is termed as motivational factors, and the latter is hygiene factors (Herzberg et al., 1959; Herzberg, 1966, 1968). Building on two-factor theory, we propose that some products would make consumers feel satisfied, whereas other products could only make consumers not to feel dissatisfied. Specifically, consumers would feel dissatisfied if they are lack of “fundamental products” or “hygiene products,” and they would not feel dissatisfied when they obtain these kinds of products. In contrast, consumers would feel satisfied if they get the “motivational products.” In another word, providing fundamental or hygiene products would only guarantee the “no dissatisfaction” situation rather than increase satisfaction of individuals, whereas obtaining motivational products would satisfy consumers.

Accordingly, we hypothesize that while consuming fundamental products would not help relieve stress, and the repression of fundamental consumption would evoke consumer stress, as they cannot live without must-have products. The repression of hygiene consumption would lead to psychological distress as well, since consumers would be dissatisfied if they could not obtain enough hygiene products. Stress would be reduced by obtaining hygiene products again, playing the role of a problem-focused coping strategy. In contrast, the repression of motivational consumption would not elicit stress. Consumers would simply feel no satisfaction rather than feeling dissatisfactory if purchases of motivational products were limited. However, consuming more motivational products can contribute to the release of stress, as those products would bring satisfaction to consumers, acting as an emotion-focused coping approach.

We assume that product categories are changeable depending on the type of psychological distress under consideration. For example, a product may be classified as fundamental in response to the lack of control, but at the same time, become motivational in response to the lack of freedom. In addition, the role each product plays in inducing or alleviating psychological distress varies with the situation. For example, medical care is a fundamental product during the pandemic, but it is a hygiene product otherwise.

According to the Maslow’s hierarchy of needs, food and beverages fulfill the first basic need of consumers; spending on food remains stable due to daily consumption needs. Accordingly, any change related to the consumption of food and beverages (such as a price fluctuation or supply shortage) will be considered a threat to the normal life of consumers. Hence, we propose that food and beverage is a fundamental product category based on the sense of being threatened (H_1_). The second basic need of consumers is safety. As COVID-19 continues to spread, the virus has become one of the biggest threats to the health of the consumers. If consumers cannot obtain enough medical care (consumption repression) to protect themselves and their families, their sense of being threatened would increase. Therefore, we propose that medical care is also a fundamental product based on the sense of being threatened (H_2_).

Socialization is the third basic need of consumers. People control their social lives through social activities, such as dining out and entertainment. However, consumers have had to cancel these activities during the pandemic, leading them to feel as though they have lost control of their social lives. We posit that products correlated to socializing (beauty and clothing, entertainment, and dining out) are fundamental activities based on the sense of lack of control (H_3_). Scholars have asserted that consumers take actions to restore control if they feel a lack of control (Cutright, 2012; Cutright et al., 2013). One response to the lack of control is to spend money strategically (Durante and Laran, 2016), as in purchasing products that are more useful for survival in daily life (Whitson and Galinsky, 2008; Kay and Eibach, 2013). Consumers also choose larger-sized products to gain power and control (DuBois et al., 2012). Spending on daily life items is a problem-focused action, whereas selecting larger sizes is an emotion-focused action. Hence, we propose that medical care is a hygiene product based on lack of control (H_4_), whereas furniture and appliances are motivational products based on lack of control (H_5_).

Consumers feel less freedom if they are required to stay at home instead of dining out. Compared to entertainment that can be realized through both online and offline platforms, dining can only be achieved by going out. People with fewer medical resources are less willing to go out, since they might be infected without adequate protection. Therefore, constraints on dining out or receiving medical care leads to a sense of lack of freedom. There are two ways to relieve the stress of lack of freedom: dining out again (problem-focused) or expecting to purchase other products in the future (emotion-focused). Hence, we hypothesize that medical care is a fundamental product based on the lack-of-freedom stress (H_6_). Dining out is a hygiene product based on lack of freedom (H_7_), whereas other products (food and beverage, beauty and clothing, entertainment, furniture, and appliances) become motivational products based on lack of freedom (H_8_).

### The Research Framework

**FIGURE 1:**
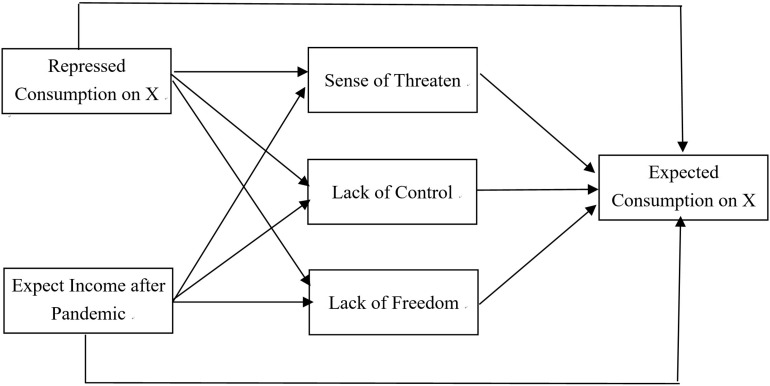
The empirical research framework.

Building on the hypothesis proposed in this study, we formulated our research framework as shown in Figure 1. As assumed, during the pandemic of COVID-19, the repressed consumption of different products (X) will directly induce different psychological distress, including the sense of threaten, lack of control, and lack of freedom. Accordingly, the psychological distress of consumers will influence their expected consumption in the future. Meanwhile, the repressed consumption on X during the pandemic will also influence the expected consumption of consumers on X after the pandemic.

Besides that, the income of the consumer plays an important role in influencing the psychology and purchase behavior of the consumer. Therefore, we also considered the expected income after the pandemic as an independent variable, influencing the psychological distress of consumers during the pandemic and the expected consumption on X after the pandemic.

## Methodology

### Data Collection

The data used in this study was collected through investigating a national questionnaire survey, and the questionnaire consisted of five parts of the consumption of consumers before and during the COVID-19 pandemic, their consumption expectation after the pandemic, and the psychological status during the pandemic. The questionnaire was delivered through Credamo, a professional data collection platform, on March 17, 2020, when the COVID-19 pandemic was considered effectively controlled in China and when Chinese consumers were gradually going back to work after nearly 1 month of staying at home.

We received 500 replies from the platform, and two participants were excluded for incomplete information (99.6% efficiency). Of the valid 498 samples, 43.8% were female, and 96.4% aged between 18 and 45 (aged 18-38: 76.1%; aged 39-45: 20.3%). About 72.5% of respondents held a bachelor’s degree or higher. The average family income per month of the samples was RMB11,370.

#### Purchase Behavior and Consumption Expectation

The purchase behavior of consumers during the pandemic was measured by family spending on six types of products in February 2020. The food and beverage refer to daily food, drinks, and condiments. Beauty and clothing include skincare products, cosmetics, clothes, and accessories. Entertainment includes online entertainment, such as membership fees for music, movie, or video-game platforms, and offline entertainment, such as tickets for museums or amusement parks. Furniture and appliance spending are self-explanatory. Dining out refers to the expenses related to eating at restaurants. Medical care includes products that can protect people from being infected by the virus, such as masks and disinfectants, and products for building immunity, such as healthcare and fitness products.

Participants were also required to compare their February spending with consumption before the pandemic on a 5-point scale (1 = significantly reduced, 5 = significantly increased) and forecast their purchase behavior after the pandemic on a 5-point scale (1 = significantly reduced, 5 = significantly increased). The results of spending comparisons were reverse-calculated and represent the extent of consumption repression the higher score represent the higher extent of consumption repression. The descriptive data of spending of consumers during the pandemic, the extent of consumption repression, and consumption expectations are shown in Table 1.

**TABLE 1:** The consumption before, during, and after the pandemic.

Products	Spending (RMB)		Repression		Expectation	
	Mean	SD	Mean	SD	Mean	SD
Food and beverage	1985.44	1838.34	2.19	1.201	3.36	1.008
Beauty and clothes	1169.07	2657.95	3.75	1.113	3.76	1.088
Entertainment	294.58	740.21	4.14	1.157	3.64	1.236
Furniture and appliances	1160.63	6105.00	3.86	1.170	3.16	1.040
Medical care	312.45	587.70	1.99	1.134	2.97	1.156
Dining out	159.54	551.22	4.46	0.972	3.84	1.198

The data show that Chinese consumers spent most of their money on products within the food and beverage category during the pandemic and was followed by the beauty and clothing category. Spending on dining out and entertainment represented only a fraction of the total consumption of consumers. Compared with the normal consumption of consumers before the pandemic, spending on dining out and entertainment were significantly repressed during the pandemic; the repression of medical care was smaller, as were both food and beverage spending and consumption. Consumers expect to increase spending the most on dining out, beauty and clothing products, and entertainment in the future.

#### Psychological Distress During the COVID-19 Pandemic

We examined psychological distress from three aspects: sense of being threatened, lack of control, and lack of freedom. The sense of being threatened was measured based on a scale proposed by Taylor et al. (2020); lack of control was measured by a scale adapted from Wallston et al. (1987) and Lachman and Weaver (1998); and lack of freedom was measured by a scale designed by Suppes (1996). All variables of psychological distress were measured on a 5-point Likert scale.

Three kinds of psychological distress were all measured with high reliability (Hair et al., 1998), as all Cronbach’s alpha values were above 0.6 (sense of threaten 0.626, three items; lack of control 0.814, three items; and lack of freedom 0.835, four items). According to the approach proposed by Fornell and Larcker (1981), the measurement of psychological distress has high convergent validity with all square roots of AVE are above 0.7 (sense of threaten 0.759, lack of control 0.857, and lack of freedom 0.824) and high discriminant validity (all square roots of AVE are much larger than their correlations).

### Data Analysis and Empirical Results

#### Data Analysis

According to the empirical framework, there are many direct and indirect causal relationships among the repressed consumption, psychological distress, and expect consumption. Therefore, structural equation modeling (SEM) was adopted in our study to estimate these different relationships. SEM as a multivariate technique, combining regression, factor analysis, and analysis of variance, is very useful to estimate interrelated dependence relationships simultaneously (MacCallum and Austin, 2000).

Based on the survey data, and using the software of AMOS 21, six SEM models were tested for the six different product categories (food and beverage, beauty and clothing, entertainment, furniture and appliances, medical care, and dining out), respectively. The empirical results were presented in Table 2.

**TABLE 2:** The empirical results of SEM models concerning different product categories.

Influencing factors		Influenced	Food and	Beauty and	Entertainment	Furniture and	Medical care	Dining out
		factors	beverage	clothing		appliances		
Repressed consumption	—>	Sense of threaten	−0.066*	0.017	0.038	–0.001	0.118***	–0.056
Repressed consumption	—>	Lack of control	–0.02	−0.172***	−0.1***	−0.153***	0.081**	−0.284***
Repressed consumption	—>	Lack of freedom	–0.044	0.137***	0.131***	0.12***	−0.147***	0.308***
Expected income	—>	Sense of threaten	0.019	0.031	0.034	0.029	0.029	0.02
Expected income	—>	Lack of control	0.079**	0.058	0.068*	0.054	0.082**	0.034
Expected income	—>	Lack of freedom	−0.074*	–0.048	–0.049	–0.045	−0.067*	–0.015
Sense of threaten	—>	Expected consumption	–0.036	–0.057	–0.02	0.048	0.025	–0.02
Lack of control	—>	Expected consumption	0.003	0.017	–0.003	0.093**	0.144***	0.004
Lack of freedom	—>	Expected consumption	0.096**	0.186***	0.167***	0.052	0.027	0.169***
Repressed consumption	—>	Expected consumption	0.091**	0.017	0.015	−0.117***	–0.025	–0.01
Expected income	—>	Expected consumption	0.198***	0.239***	0.277***	0.22***	0.047	0.255***
Goodness of model fit index		CMIN	5.569	3.012	3.905	3.329	4.071	0.344
		GFI	0.996	0.998	0.997	0.998	0.997	1.000
		CFI	0.944	0.983	0.972	0.983	0.958	1.000
		RMR	0.024	0.017	0.02	0.018	0.02	0.006
		RMSEA	0.096	0.064	0.076	0.068	0.079	0.000

RMR, root mean square residual; RMSEA, root mean square error of approximation. ***p < 0.01; **p < 0.05; *p < 0.1.

The empirical results indicated that all the proposed six SEM models provided a good fit for the data. For all the models, the goodness of fit index (GFI) and comparative fit index (CFI), two non-statistical measures in which higher value indicate a better fit, all approached 1.0, with the lowest of 0.944 and highest of 1.000. All of these meet the desired threshold of 0.90 (Hair et al., 1995). Meanwhile, all the root mean square residual (RMR) ranged from 0.006 to 0.024, meeting the acceptable threshold of 0.05, and the lower the better, and all the root mean square error of approximation (RMSEA) ranged from 0.000 to 0.096, also meet the acceptable threshold of 0.1 (Hair et al., 1995).

The model demonstrated that the repressed consumption of different product would cause different psychological distress, and accordingly these psychological distress would induce different consumption expectation. For the model of food and beverage, the repressed consumption on food and beverage during the pandemic would release the sense of threaten (path coefficient = −0.066, *p* < 0.1); however, the lack of freedom will increase the expected consumption on food and beverage in the future (path coefficient = 0.096, *p* < 0.05). For the model of beauty and clothing, the repressed consumption during the pandemic would help to release the lack of control (path coefficient = −0.172, *p* < 0.01) but induce the lack of freedom (path coefficient = 0.137, *p* < 0.01); meanwhile, the lack of freedom would also increase the expected consumption on beauty and clothing in the future (path coefficient = 0.186, *p* < 0.01). For the model of entertainment, the result was quite the same as the beauty and clothing, the repressed consumption during the pandemic would help to release the lack of control (path coefficient = −0.1, *p* < 0.01) but induce the lack of freedom (path coefficient = 0.131, *p* < 0.01); meanwhile, the lack of freedom would also increase the expected consumption on entertainment in the future (path coefficient = 0.167, *p* < 0.01). For the model of furniture and appliances, the repressed consumption during the pandemic would help to release the lack of control (path coefficient = −0.153, *p* < 0.01) but induce the lack of freedom (path coefficient = 0.12, *p* < 0.01); meanwhile, the lack of control would also increase the expected consumption in the future (path coefficient = 0.093, *p* < 0.01). For the model of medical care, the repressed consumption during the pandemic would induce the sense of threaten (path coefficient = 0.118, *p* < 0.01) and lack of control (path coefficient = 0.081, *p* < 0.05) but could help to release the lack of freedom (path coefficient = −0.147, *p* < 0.01). For the model of dining out, the result was also similar with the beauty, clothing, and entertainment, the repressed consumption would help to release the lack of control (path coefficient = −0.284, *p* < 0.01) but induce the lack of freedom (path coefficient = 0.308, *p* < 0.01); meanwhile, the lack of freedom would increase the expected consumption on dining out in the future (path coefficient = 0.169, *p* < 0.01).

#### Influence of Repressed Consumption on Psychological Distress

The empirical results confirmed our basic premise that different types of consumption repression led to different types of psychological distress. Specifically, the sense of being threatened is negatively affected by the repression of food and beverage consumption, but positively affected by the repression of medical care. This result indicates that increased food purchases may release the sense of being threatened feeling in consumers during the pandemic, whereas repression of medical care spending would increase the sense of being threatened feeling in the consumers.

Lack of control is negatively influenced by the repression of socialization-related activities and products, such as items in the beauty and clothing category, entertainment and dining out, but positively affected by the repression of medical care consumption. The results indicate that reducing the social activities and socialization-related consumptions during the pandemic may help the consumers to gain a sense of control; meanwhile, the decrease of spending on medical care will induce the consumers to lose the sense of control during the pandemic.

However, the relationship between consumption and lack of freedom shows an opposite situation compared with the lack of control. The lack of freedom of consumers is positively influenced by the repression of socialization-related activities and products, such as items in the beauty and clothing category, entertainment and dining out, but negatively influenced by the repression of medical care consumption. After the outbreak of the COVID-19 pandemic, many governments applied strict isolation polices and banned many social activities. The socialization-related consumption of consumers was reduced leading to the increasing feeling of lack of freedom. Meanwhile, the increased spending on medical care, such as masks, would lead to a rising sense of lack of freedom.

#### Influence of Psychological Distress on Consumption Expectation

The six SEM models can also present how expectations regarding future spending relieve psychological distress during the pandemic. The empirical results show that the purchase of different products will reduce their corresponding psychological distress caused by consumption patterns during the COVID-19 pandemic. Specifically, consumption expectations in relation to food, beauty, clothing, entertainment, and dining out are positively influenced by lack of freedom during the pandemic. Meanwhile, the consumption expectations in relation to furniture and appliances and medical care are positively influenced by a lack of control during the pandemic. However, the sense of being threatened could not be relieved by any consumption expectation. During the COVID-19 pandemic, the sense of being threatened to feel in consumers was elicited by both the actual changes in daily life and possible changes to health status, with the latter dominating. Hence, the sense of being threatened would not disappear until the pandemic ended; no expectations of consumption could reduce the sense of being threatened.

Besides that, our results also demonstrate that all consumption expectations except those for medical care are positively influenced by future income expectations. If consumers expect their incomes will grow in the future, they will increase their purchases of all types of products.

## Discussion

**FIGURE 2:**
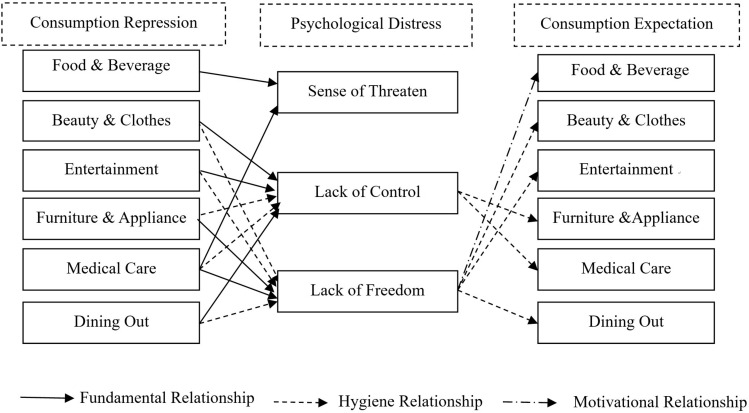
The relationship between consumption repression, psychological distress and consumption expectation for different products.

Our study explored consumption repression during the COVID-19 pandemic and consumption expectation after the pandemic for various product categories. We investigated how consumption repression induced three types of psychological distress, and how that stress was alleviated by the consumption expectation in the future after the pandemic. Figure 2 illustrates the results of our framework. Specially, we found that repressed consumption of fundamental products (such as food and beverage, and medical care) would result in an increasing sense of threaten. But the consumption expectation of these products could not alleviate the sense of threaten. In terms of lack of control, socialization-related products such as those pertaining to self-image, entertainment, and social activities such as dining out, are fundamental products, because the consumption of these may increase the risk of being infected. However, repressed consumption of these kinds of products could reduce the sense of lack of control, but the consumption expectation of them could not improve the sense of lack of control. In contrast, furniture and appliance act as hygiene products for the sense of lack of control. The repression could result in a lack of control, which could also be alleviated by the consumption expectation. In terms of lack of freedom, furniture and appliance, and medical care act as fundamental products; food, self-image, entertainment, and social activities act as motivational products. Consumers feel a lack of freedom if they cannot dine out or obtain enough medical care to protect themselves when they go out. This feeling of lack of freedom decreases if consumers can dine out again, a problem-focused solution, or if they expect to purchase other food or self-image and entertainment products to make themselves forget the current feeling, an emotion-focused approach.

Our research made three contributions to current research. Compared to previous research that mainly focused on one type of products (Grashuis et al., 2020; Kim, 2020), our research combined various product types together, and we categorize the products and services into three types: functional, hygiene, and motivational, based on Maslow’s theory of needs. It is important because we provide a comprehensive way to study consumption and the needs that motivate consumers to purchase.

Moreover, taking the perspective of Herzberg’s two-factor theory, we highlighted that fundamental and hygiene consumption could only guarantee “no satisfaction” of individuals, yet the repression of these kinds of consumption could elicit the corresponding stress, but the recovery of this consumption could not alleviate the stress. In contrast, motivational consumption could increase the satisfaction of individuals. Hence, the repression of motivational consumption would not lead to the stress, but the recovery of motivational consumption is effective to reduce stress. Different from previous research that agreed with the negative influence of repressed consumption on psychological distress (Ma et al., 2019; Chodkiewicz et al., 2020; Chenarides et al., 2021), our research found that depression of some certain types of consumption could even alleviate the stress during the period of COVID-19 outbreak. The interesting findings provide new insight for future studies to re-understand the relationship between consumption and psychological distress.

Third, building on the theory of coping strategies, our research confirms the effectiveness of consumption in reducing corresponding stress. The findings shed light on both emotion-focused and problem-focused coping from the perspective of consumption. We also emphasized the importance of matching consumption with different stress. Future studies could continue the research to compare the influence of fundamental, hygiene, and motivational consumption in reducing stress.

Our research highlighted the correlation between consumption and psychological distress by categorizing the products into three categories. The conclusion was obtained based on the data during the outbreak of COVID-19. Since it is a special period, one may argue that if the proposed matching between consumption and psychological distress can be applied to other regular occasions. Although previous literature has provided some scattered evidence, we admitted that further research needs to be conducted to test our comprehensive framework after the pandemic.

## Conclusion

Utilizing consumption data and psychological data obtained during the COVID-19 pandemic in China, we found that consumption repression leads to different psychological distress, but the different psychological distress can be reduced by different consumption expectations. Specifically, we divided products into fundamental, hygiene, and motivational categories. We confirmed that constraint in the consumption of relatively fundamental or hygiene products would elicit a sense of being threatened, a sense of lack of control, and/or a sense of lack of freedom, whereas the expectation of purchasing hygiene or motivational products can relieve the corresponding stress. The future studies could continue to examine the actual purchase behavior after the pandemic to further test our proposition. Marketers and policymakers will benefit from the findings by managing the product supply during and after the COVID-19 pandemic, and in future relevant social emergencies.

## Data Availability Statement

The raw data supporting the conclusions of this article will be made available by the authors, without undue reservation.

## Ethics Statement

Ethical review and approval was not required for the study on human participants in accordance with the local legislation and institutional requirements. The patients/participants provided their written informed consent to participate in this study.

## Author Contributions

YW and SC performed the material preparation, data collection, and analysis. YW and YL wrote the first draft of the manuscript. All authors contributed to the study conception and design, commented on previous versions of the manuscript, read, and approved the final manuscript.

## Conflict of Interest

The authors declare that the research was conducted in the absence of any commercial or financial relationships that could be construed as a potential conflict of interest.

## Publisher’s Note

All claims expressed in this article are solely those of the authors and do not necessarily represent those of their affiliated organizations, or those of the publisher, the editors and the reviewers. Any product that may be evaluated in this article, or claim that may be made by its manufacturer, is not guaranteed or endorsed by the publisher.
